# Isinglass as an Alternative Biopolymer Membrane for Green Electrochemical Devices: Initial Studies of Application in Electric Double-Layer Capacitors and Future Perspectives

**DOI:** 10.3390/polym15173557

**Published:** 2023-08-26

**Authors:** Paweł Jeżowski, Przemysław Łukasz Kowalczewski

**Affiliations:** 1 Institute of Chemistry and Technical Electrochemistry, Poznan University of Technology, 4 Berdychowo St., 60-965 Poznań, Poland; 2 Department of Food Technology of Plant Origin, Poznań University of Life Sciences, 31 Wojska Polskiego St., 60-624 Poznań, Poland

**Keywords:** biopolymer, membrane, energy storage, green chemistry, EDLC

## Abstract

The presented work discusses in detail the preparation of a cheap and environmentally friendly biopolymer membrane from isinglass and its physicochemical characterisation. One of the possible uses of the obtained membrane can be as a separator between electrodes in novel green electrochemical devices as in, for example, electric double-layer capacitors (EDLCs). The functionality of the mentioned membrane was investigated and demonstrated by classical electrochemical techniques such as cyclic voltammetry (CV), galvanostatic cycling with potential limitation (GCPL), and electrochemical impedance spectroscopy (EIS). The obtained values of capacitance (approximately 30 F g^−1^) and resistance (approximately. 3 Ohms), as well as the longevity of the EDLC during electrochemical floating at a voltage of 1.6 V (more than 200 h), show that the proposed biopolymer membrane could be an interesting alternative among the more environmentally friendly energy storage devices, while additionally it could be more economically justified.

## 1. Introduction

Recent years have been dominated by synthetic polymers; from everyday-use simple appliances to highly sophisticated equipment, you can be certain to find it in your nearest surroundings. Since 2004, more than 4.6 billion tons of plastics have been produced [1]. The increasing use of synthetic polymers causes a worldwide dilemma due to pollution [2]. Biopolymers could substitute for synthetic ones in any possible field; for example, in electrochemistry, all devices are composed of two electrodes that are separated by a porous membrane that allows for the flow of ions. Usually, the membrane is made of materials such as glass fibre, which has potential cancerogenic properties [3], nafion, which is classified as a hazardous material, or poly(propylene), which is responsible for the release of microplastic due to degradation [4]. Biopolymers are currently widely investigated as a possible way to exchange problematic and hazardous materials [5]. The use of cellulose, chitin [6,7], chitosan [8,9], dextrin [10,11], dextran [12,13], agar [14,15], lignin [16,17], and other biopolymers [18,19,20,21,22] have been reported in the literature, with their promising use in energy storage devices such as lithium-ion batteries (LIBs) [23,24,25] or electric double-layer capacitors (EDLCs) [26,27,28]. Biopolymers can be used for almost any aspect of electrode construction, from an electrode material binder [8], to conductor glue for improved adhesion and electrical conductivity [29], to an electrolyte [30], and even for preparation of active material responsible for energy storage mechanisms [31,32]. Most of the studies on the use of biopolymer membranes in EDLCs are focused on the use of aqueous electrolytes and can be summarised in Table 1.

However, the preparation of biopolymer membranes presented in the literature can be time-consuming and can even take up to 6 h at an elevated temperature of 50 °C [40], can be complicated in execution [41], or can simply be costly [36] due to the use of additional chemicals that cannot be considered environmentally friendly or green. In the mentioned studies, the use of potassium iodide or glutaraldehyde, which are considered toxic to the environment as well as human beings, is rather in opposition to the main goal of green biopolymers, which is their environmental friendliness or at least their benign character. Furthermore, price is often omitted in scientific studies, but from an economical point of view, the use of 1-butyl-3-methylimidazolium acetate (close to 1500 USD per 1 kg) could be hard to justify for a bigger-scale preparation of a sustainable biopolymer membrane for any application. Furthermore, the use of biopolymer membranes allows for the mitigation of a possible spill of liquid electrolyte from the electrochemical cell in case of cell body damage, as most of the electrolyte is stored in the membrane volume [42]. However, the use of liquid electrolytes is one of the most-used approaches because of the simplified construction and automatization of cell assembly on a larger scale. Today, the reuse of wastes from fish production, including isinglass, presents a promising avenue for addressing both environmental and technological challenges. Isinglass, a protein-rich substance obtained from fish bladders, is commonly used in the beer and wine industry as a fining agent [43,44,45]. However, its potential extends far beyond this traditional application. In the realm of electrochemistry, isinglass can serve as a valuable precursor for the development of advanced alternative biopolymer membranes for green electrochemical devices and can be employed as electrodes in various energy storage and conversion devices, such as supercapacitors and fuel cells. Utilising fish waste-derived isinglass as a membrane material not only reduces environmental burdens by reusing otherwise discarded by-products but also contributes to the advancement of eco-friendly and sustainable electrochemical technologies. This interdisciplinary approach holds great promise in creating a more circular economy while fostering advances in renewable energy and green technology sectors.

For the mentioned reasons, in this research, we decided to prepare a cost-effective and green biopolymer membrane that could be successfully incorporated into energy storage devices like, for example, EDLCs. An isinglass membrane can be successfully prepared by heating it with a small amount of deionized water. Furthermore, isinglass possesses flexible properties that can be used in the construction of novel wearable energy storage systems [46]; for example, different types of smartwatches, which are becoming more popular each day, not only among young generations but also for the elderly, as they can be used for heart monitoring. These electronics could implement energy storage cells inside the band and the flexible solar cell outside the band to maximize their longevity.

## 2. Materials and Methods

### 2.1. Preparation of Biopolymer Membrane

**Figure 1:**
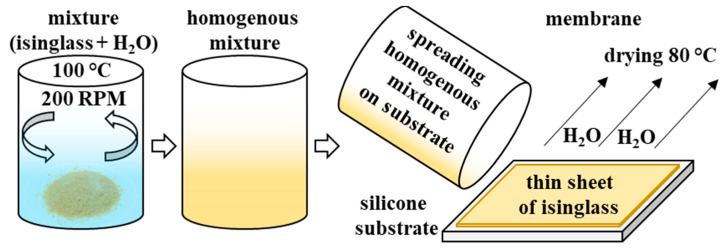
Schematic illustration of the isinglass membrane preparation process.

Isinglass (1 g, protein content 75–80%, humidity 10–12%, ashes 8–12%, Kremer Pigmente GmbH + Co. KG, Aichstetten, Germany) was placed in a stainless-steel beaker with 10 mL of distilled water. The heterogeneous mixture was heated (100 °C) and stirred (200 RPM) until excess solvent evaporated and a homogenous viscous yellowish mixture was obtained. It was then casted on a silicone board and spread across its surface to obtain a biopolymer membrane as thin as possible and then was placed in a universal laboratory convection oven heated up to 80 °C for approximately 1 h (UN30, Memmert GmbH + Co. KG, Schwabach, Germany). Once the membrane was dry, its thickness was measured (approximately 150 μm) and it was introduced into the aqueous electrolyte solution of 1 mol L^−1^ Li_2_SO_4_ and stored in such conditions until the separators were cut with hollow punchers (diameter 12 mm). The preparation of the isinglass membrane is summarised in Figure 1.

### 2.2. Electrochemical Cell Preparation

The electrodes for EDLCs were cut with hollow punchers (diameter 10 mm) of Kynol ACC-507-20 carbon cloth with an average mass of each electrode of approximately 8 mg ± 1 mg. The electrodes were introduced to the Swagelok laboratory electrochemical cell with stainless steel (316L) current collectors coated with conductive glue [29] and separated by a prepared biopolymer membrane (described in Section 2.1) or by a glass microfiber disk (diameter 12 mm and thickness 260 μm, Whatman^®^ GF/A, Sigma-Aldrich, Darmstadt, Germany). Finally, each electrode was soaked with approximately 100 μL electrolyte. Additionally, to present the flexible properties of the isinglass membrane, porotype pouch cells were assembled to highlight excellent performance when an external force is applied to the cell body during electrochemical testing.

### 2.3. Electrochemical Testing Schedule

Cyclic voltammetry (CV) was performed at various voltage windows from 1.0 to 2.0 V and at a constant scanning rate of 5 mV s^−1^ to determine the maximum operational voltage for the rest of the electrochemical investigations, according to the calculation of the S-value presented by Weingarth et al. [47]. Later, several scanning rates were used from 1.0 to 100 mV s^−1^ at 1.6 V voltage to observe qualitative changes in the charge accumulation. Galvanostatic cycling with potential limitation (GCPL) was carried out at a voltage of 1.6 V with different current densities from 100 mA g^−1^ to 5000 mA g^−1^ to quantitatively establish the capacitance, energy, and power of the electrochemical cells. Furthermore, the constant power (CP) technique was implemented to schedule electrochemical investigations to adequately calculate the energy and power of electrochemical cells [48]. Potentiostatic electrochemical impedance spectroscopy (PEIS) was performed to measure the internal resistance (IR) of electrochemical cells (composed of equivalent series resistance (ESR) and equivalent distributed resistance (EDR)) in the range of frequencies from 1 mHz to 100 kHz at the sinusoidal amplitude of the input signal 5 mV. Change in the resistance upon floating was measured by the Current Interrupted (CI) technique, where small pulses of current separated by rest periods allows one to estimate the internal resistance of the device. The Electrochemical Floating Test (EFT) allowed for accelerated ageing of the electrochemical cell by holding the maximum voltage of the electrochemical cell for an estimated time period of 2 h several times and establishing its stability during the overall time of the test. All valueswere calculated per mass of both electrodes. The equipment used for electrochemical investigations was VMP3 (BioLogic, Seyssinet-Pariset, France) and EC-Lab software version 11.32 (BioLogic, Seyssinet-Pariset, France) to process all acquired data.

### 2.4. Raman Spectroscopy

To observe the structure and texture of the sample, a DXR3xi Raman Imaging Microscope (Thermo Fisher Scientific Inc., Waltham, MA, USA) was used. Photographic images were taken at the different magnifications ×10 and ×50, and the Raman spectra were taken using a laser at 532 nm wavelength with power of 10 mW. The spectrum was acquired in the range of 200 to 3500 cm^−1^ wavenumber at magnification ×50.

## 3. Results and Discussion

**Figure 2:**
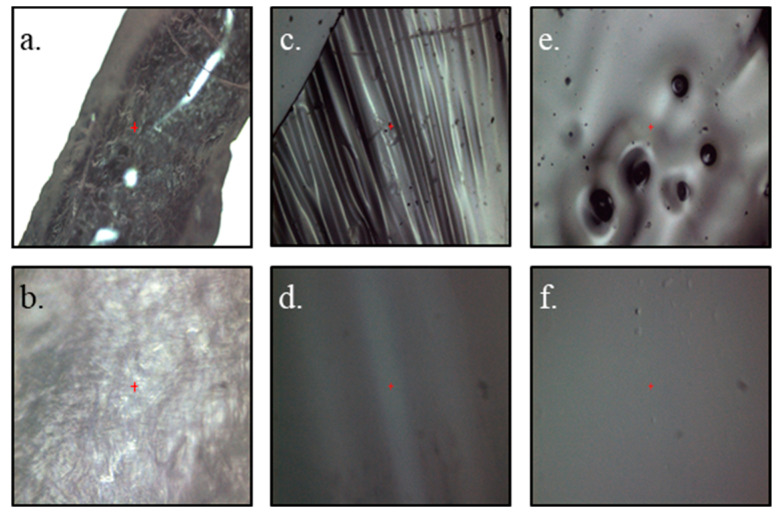
Microscopic images at magnification ×10 (**a**,**c**,**e**) and ×50 (**b**,**d**,**f**) for as-received isinglass particles (**a**,**b**), dry biopolymer isinglass membrane (**c**,**d**), and membrane soaked with electrolyte (**e**,**f**). Red plus sign is a central point of take image.

The as-received isinglass particles (Figure 2a,b) were observed under a microscope to see the changes that occurred after the preparation of the biopolymer membrane (Figure 2c,d). As seen, the texture of the substrate and the product are completely different from each other; substrate particles are rough and uneven. The dried biopolymer membrane has an intriguing drapery-like texture and the material itself is flexible but brittle. The pictures of the membrane in Figure 2e,f were taken after the drop of electrolyte (1 mol L^−1^ Li_2_SO_4_ in H_2_O) was in direct contact with the biopolymer membrane. The texture of the biopolymer membrane flattened and evened out. Additionally, the porous structure of the membrane began to be visible. Moreover, the membrane started to be elastic, flexible, and easily adhered to the surface of the electrode.

**Figure 3:**
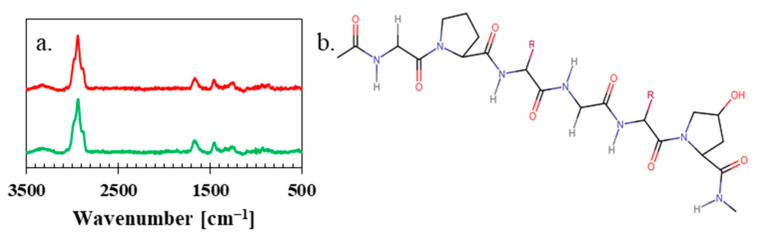
(**a**) Raman spectra of isinglass particles (upper red curve) and dry biopolymer isinglass membrane (lower green curve) and (**b**) chemical structure of the main chemical component of isinglass.

Raman spectra proved that the textural changes in the material had no impact on the structure, as isinglass particles (upper red curve) and the dry biopolymer isinglass membrane (lower green curve) have almost identical visible vibrations in Figure 3a, which are coming from amide I and amide III, which are sensitive to the changes in the structure. These vibrations are visible near the spectral range from 1200–1750 cm^−1^. Bands visible near 1200–1400 cm^−1^ can be assigned to the following C-C stretching and N-H in-plane bending vibrations. Bands in the range 1600–1700 cm^−1^ are usually related to the C = O stretching vibrations of the peptide bond and N-H bending vibrations. Furthermore, the most visible bands in the wavenumber range 2800–3200 cm^−1^ are -CH_2_ stretching vibrations [49]. Finally, the noticeable bump above 3200 cm^−1^ comes from the stretching vibrations of the -OH group. This shows that the proposed isinglass is mainly composed of I-type collagen [50], presented in Figure 3b.

**Figure 4:**
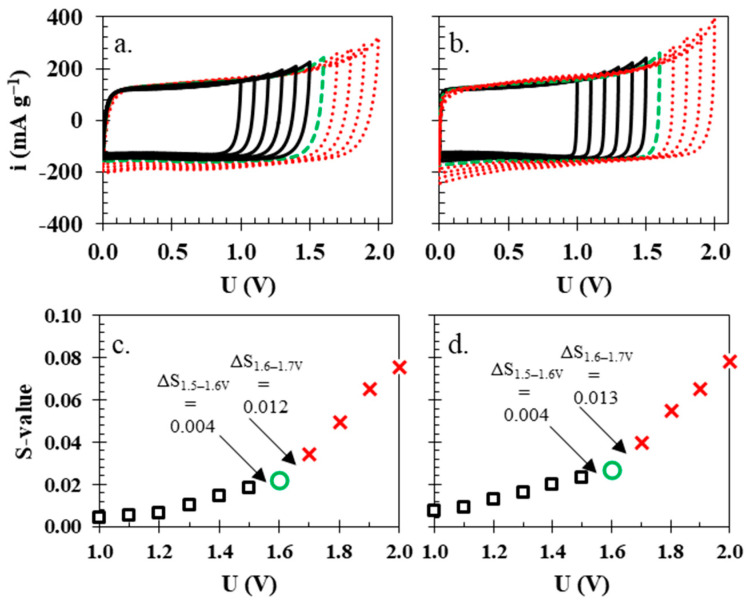
Cyclic voltammograms of electrochemical cells with (**a**) isinglass membrane and (**b**) glass fibre separator. The S-values calculated for cells with (**c**) biopolymer membrane and (**d**) glass fibre separator.

The isinglass membrane was introduced into the electrochemical cell with two carbon electrodes and 1 mol L^−1^ Li_2_SO_4_. Cyclic voltammetry studies at 5 mV s^−1^ and with an increase in operational voltage from 1.0 to 2.0 V are presented in Figure 4a,b. The shape of both of the cyclic voltammograms is nearly rectangular, which implies that the energy storage mechanism is purely capacitive. In the case of isinglass, the rectangular shape of cyclic voltammograms was almost unaffected by the increased voltage; on the other hand, the electrochemical cell with the glass fibre separator presented a noticeable rise in anodic and cathodic current, especially above the value ca. 1.6 V. To correctly estimate the highest possible operational voltage for each cell, the S-value was calculated [47] at every investigated voltage. The S-value is calculated from the CV data. Firstly, the surface area is integrated above (anodic) and below (cathodic) current of 0 mA g^−1^ (y axis) at each voltage. Then, the ratio of integral values for anodic and cathodic current is calculated, which is called the S-value (Figure 4c,d). Finally, the difference between adjacent S-values is calculated, and if the value Δ > 0.005, then it is the maximum operational voltage. For the electrochemical cell with a glass fibre separator, the maximum voltage was 1.6 V since the difference in the S-values between 1.6 and 1.7 V was greater than 0.005 (0.013), while in the case of the isinglass membrane, the difference between the S-values was 0.012. For comparative purposes, both cells were limited to an operating voltage of 1.6 V, similar to other reports [30].

**Figure 5:**
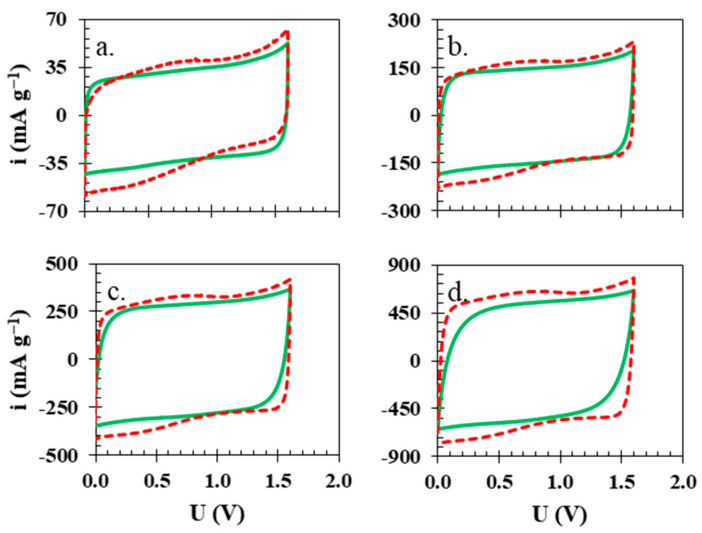
Cyclic voltammograms for electrochemical cells with isinglass membrane (solid green line) and glass fibre separator (red dashed line) at (**a**) 1, (**b**) 5, (**c**) 10, and (**d**) 20 mV s^−1^, voltage 1.6 V.

At lower scanning rates of 1 and 5 mV s^−1^ (Figure 5a,b, respectively), both electrochemical cells with different separators (solid green line—isinglass biopolymer membrane, red dashed line—glass fibre) presented a similar electrochemical behaviour. Still, the shape of voltammograms for the cell with a biopolymer membrane presents a more rectangular shape without any noticeable increases in the anodic nor the cathodic current, which is in accordance with observations from previous experiments. A noticeable difference between the two separators was observed when higher scanning rates were applied. At 10 mV s^−1^ (Figure 5c), the rectangular shape of the biopolymer membrane starts to be less rectangular, and at 20 mV s^−1^ (Figure 5d), it shows an even more resistive characteristic—due to worse charge propagation—than in the electrochemical cell with the glass fibre separator.

**Figure 6:**
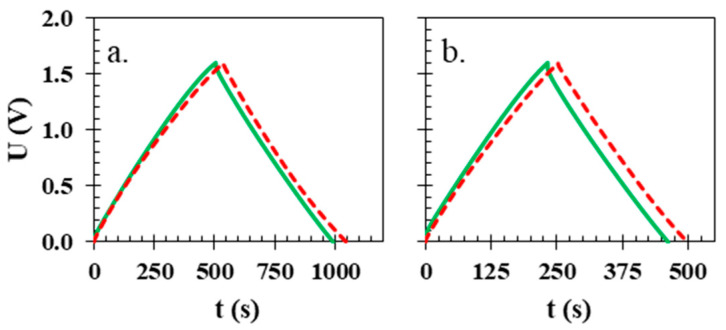

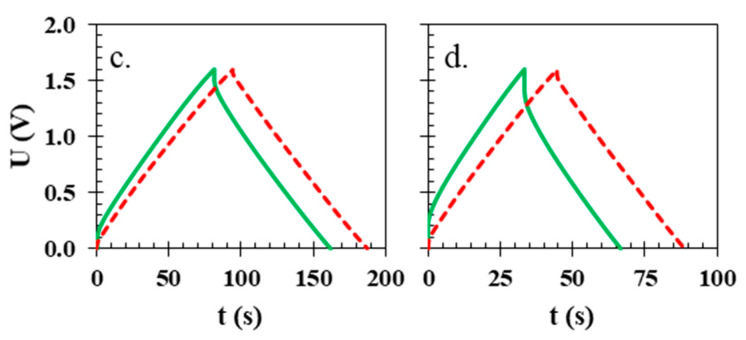
Galvanostatic charge/discharge profiles of electrochemical cells with different types of separators the biopolymer membrane (solid green line) and glass fibre disk (red dashed line) at different current densities (**a**) 100, (**b**) 200, (**c**) 500 and (**d**) 1000 mA g^−1^.

Similar to the CV results, the charge/discharge profiles indicate that the attainable capacitance for both systems is similar and near 30 F g^−1^, where both cells—the one with the glass fibre separator (red dashed lines) and the one with the isinglass (green solid lines)—reached the same value of 28 F g^−1^ at current density 100 mA g^−1^ (Figure 6a). While at the higher current densities of 200 and 500 mA g^−1^ (Figure 6b,c, respectively), the discrepancy between the two cells starts to be present due to the difference in their internal resistance; at the highest current density presented, 1000 mA g^−1^ (Figure 6d), the capacitance of the electrochemical cell with the glass fibre separator reaches ca. 27 F g^−1^, and the isinglass separator achieves only 20 F g^−1^. Increasing the current density highlights resistance differences in the case of the electrochemical cell with the biopolymer membrane and the one with the glass fibre separator.

**Figure 7:**
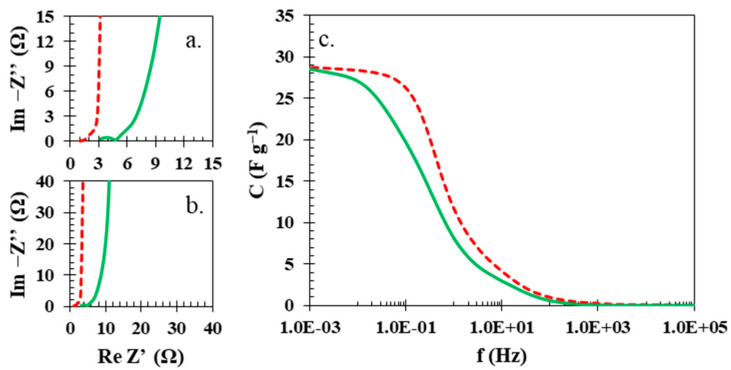
(**a**) Nyquist plots of electrochemical cells with biopolymer membrane (solid green line) and glass fibre separator (red dashed line) and (**b**) the magnification of the ESR and EDR region and (**c**) the characteristic of capacitance versus frequency.

The visible resistance discrepancy between the electrochemical cell with the biopolymer membrane and the glass fibre separator was quantitatively examined by potentiostatic electrochemical impedance spectroscopy (PEIS) (Figure 7a–c). Values of equivalent series resistance (ESR) and equivalent distributed resistance (EDR) for the electrochemical cell with the glass fibre separator (Figure 7a,b, red dashed line) are 1 and 3 Ohms, respectively. However, in the case of the electrochemical cell with the biopolymer membrane (Figure 7a,b, green solid line), these values were higher; ESR was 3 Ohms and EDR was 7 Ohms. Furthermore, the calculated values of capacitance vs. frequency proved what was also observed in the cyclic voltammetry and galvanostatic charging/discharging, that the biopolymer membrane can be successfully applied when milder conditions are applied to the electrochemical cell with a biopolymer membrane as the separator.

**Figure 8:**
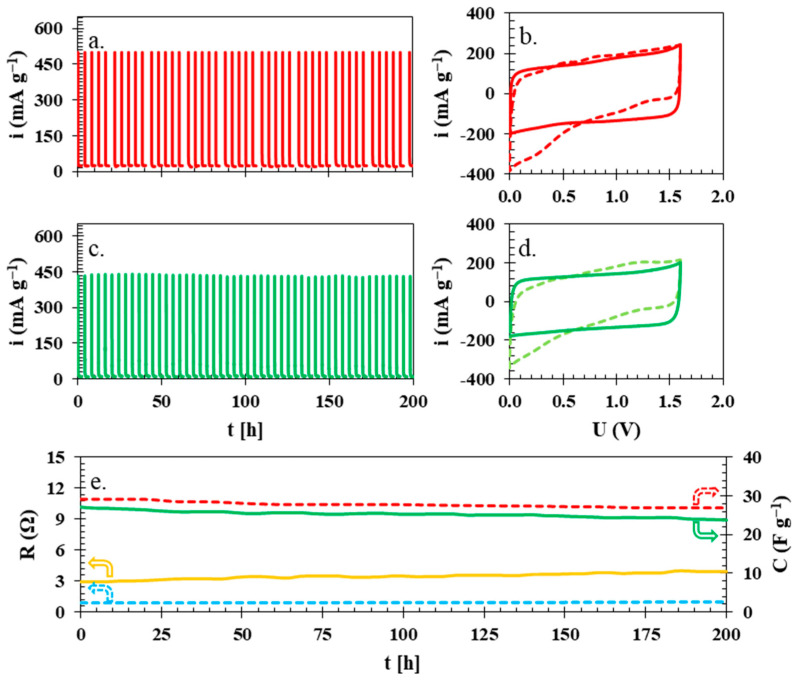
Leakage current for an electrochemical cell with (**a**) glass fibre separator and (**c**) biopolymer membrane. CV plots for electrochemical cells with (**b**) glass fibre and (**d**) isinglass separator before (solid lines) and after (dashed lines) 200 h of experiment time. (**e**) Change in capacitance (upper lines) and resistance (lower lines) during the initial 200 h of electrochemical testing of the cells with isinglass membrane (solid lines) and glass fibre separator (dashed lines).

Once all of the basic electrochemical experiments were finished, it was necessary to establish the end-of-life criterion (80% of initial capacitance or a two-fold increase in resistance). Figure 8a,c present the leakage current profile for the holding time at the maximum voltage of 2 h separated by ca. periods of 2 h, during which additional CVs were performed to present qualitative changes in CV curves (Figure 8b,d), as well as GCPL and CI experiments to quantitatively estimate capacitance and resistance, presented in Figure 8e. This 4 h time period can be considered one full cycle, which means that during the experiment presented in Figure 8a,c, the total time of holding at maximum voltage was 100 h. The data from the initial 200 h of the electrochemical technique were insufficient to establish the failure of the electrochemical cells, and it was repeated until the end-of-life criterion (80% capacitance), which was reached after 362 h of holding time at the maximum voltage of 1.6 V (the electrochemical cell with the glass fibre separator reached almost identical end-of-life criterion at 378 h). After 200 h of technique time, it was already noticeable that the shape of CVs (Figure 8b,d) had deteriorated from the typical rectangular shape, which indicates partial oxidation and reduction of electrodes, which are identical for both electrochemical cells and are similar to observations reported elsewhere [51]. During the overall time of the experiment, the capacitance of the electrochemical cell with the isinglass membrane was 27 to 24 F g^−1^, while the resistance increased from 2.9 to 4.0 Ohms; in comparison, the electrochemical cell with the capacitance of the glass fibre separator fell from 29 to 27 F g^−1^ and the resistance increased from 1.0 to 1.3 Ohm, which means the overall drop in capacitance and increase in resistance are similar in both of the cases. The leakage current data indicated that the amount of current necessary to sustain the maximum voltage during the hold period was 10 mA g^−1^ in the case of the biopolymer membrane, and for the glass fibre separator, it was 23 mA g^−1^, which can be attributed to the dielectric properties of the isinglass membrane.

**Figure 9:**
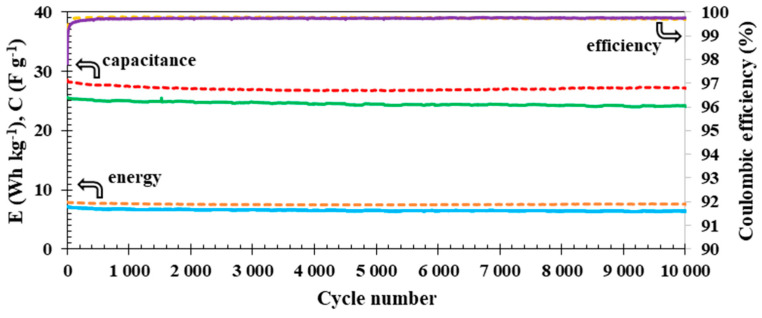
Cyclic life of the electrochemical cell with glass fibre separator (dashed lines) and biopolymer membrane (solid lines). The upper lines represent the Coulombic efficiencies of electrochemical cells, the middle lines represent the capacitance values, and, finally, the lower lines represent the specific energy data.

In addition, both electrochemical devices were subjected to electrochemical tests to establish their cyclic life (Figure 9) by continuous charge/discharge at a current density of 0.1 A g^−1^ within the voltage range of 0.0 to 1.6 V. Changes in capacitance and specific energy values during 10,000 cycles are very similar for both electrochemical cells with the isinglass membrane (solid lines) and the glass fibre separator (dashed lines). In the case of the glass fibre separator, there was a 4% decrease in the capacitance (dashed middle red line) value after 10,000 cycles, and the Coulombic efficiency was nearly 99.6% (dashed yellow upper line). For the electrochemical cell with the isinglass membrane, there was a noticeable 5% drop in the capacitance compared to the initial value (26 F g^−1^ ), while the Coulombic efficiency was ca. 99.7%. It is interesting that the initial 3% of the drop in the capacitance value was observed during the first 1000 cycles for the electrochemical cell with the isinglass membrane and the glass fibre separator. Afterwards, between cycle numbers 1000 and 10,000, the drop in capacitance value is smaller, ca. 2%. The observable capacitance in the case of electrochemical cells with a glass fibre separator and a biopolymer membrane appears to stabilise afterward. This observation is probably related to the initial penetration of the porous structure of the electrodes with the electrolyte solution and the ion entrapment inside of the carbon material.

**Figure 10:**
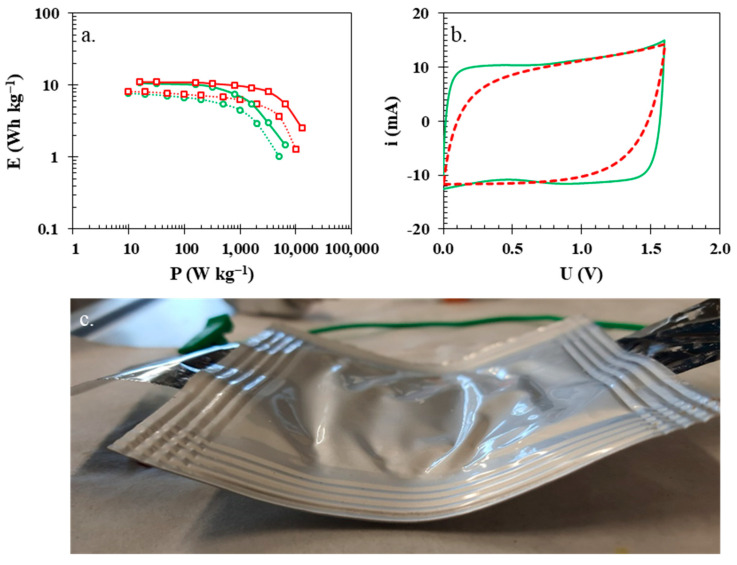
(**a**) Ragone plot of calculated energy and power based on GCPL (solid lines) and CP (dotted lines) for an electrochemical cell with a glass fibre separator (red lines with square markers) and an isinglass membrane (green lines with circle markers). (**b**) CVs of the electrochemical cells with an isinglass membrane (solid green line) and a glass fibre separator (solid red line) examined in (**c**) a prototype pouch cell.

Finally, specific values of energy and power values were calculated based on galvanostatic charge–discharge profiles, as well as the constant power discharge technique (Figure 10a), because galvanostatic experiments can be prone to overestimation of data [48]. The specific energy and specific power values for both electrochemical cells are almost identical up to the specific power value of 2 kW kg^−1^, when the higher resistance of the electrochemical cell with the isinglass membrane starts to hinder the energy of said cell (3 Wh kg^−1^). The energy of the electrochemical cell with the glass fibre separator (5 Wh kg^−1^) at higher values of specific power is even more noticeable. The presented data clearly show the better overall electrochemical performance of the electrochemical cell with the glass fibre separator at higher current loads. The isinglass possesses one intrinsic property that glass fibre separators do not have, which is flexibility. To compare both dielectrics, pouch cell prototypes were assembled and tested using cyclic voltammetry at a scanning rate of 5 mV s^−1^ up to a voltage of 1.6 V (Figure 10b) when an external force was applied to the pouch cell. Electrochemical cells were permanently bent during electrochemical testing (Figure 10c) to observe the current response when the external force was applied to the cell. As seen, the pouch cell with the biopolymer membrane (green solid line) still retains the rectangular shape characteristic for uninterrupted charge propagation, while the electrochemical characteristic of the cell with glass fibre is noticeably impacted by the applied external force. The difference between the glass fibre and the isinglass biopolymer membrane is in their structure. Although the glass fibre is made of tiny rod-shaped structures that are excellent for static applications, their flexibility is limited, and they are prone to separate from each other and increase overall resistance.

## 4. Conclusions

The obtained isinglass membranes were successfully implemented in green energy storage devices such as EDLCs. In our opinion, the isinglass membrane is an interesting alternative to the data reported so far, due to its relatively high operating voltage of 1.6 V, good longevity of 10,000 cycles (or at least 200 h of floating), and last but not least, the flexibility proven by the prototype pouch cell that we were able to prepare. Moreover, the preparation of the biopolymer membrane does not require any hazardous chemicals and is easy and fast. The capacitance, energy, and power of the assembled EDLC with a biopolymer membrane and a glass fibre separator are close to each other under milder conditions, and they are similar. The capacitance near 28 F g^−1^, a resistance of 3 Ohms, and an energy of ca. 9 Wh kg^−1^ at power 1 kW kg^−1^ are close to other results that can be found in the literature. However, the overall performance of isinglass still needs additional improvement to reach lower values of internal resistance, as this is the limiting factor and one of the biggest drawbacks of the presented idea. To mitigate the mentioned issues, we are already investigating several possible modifications to the biopolymer membrane construction and additives, which could give a promising improvement in ion transfer throughout the membrane and lower the overall internal resistance of the device, even at stationary conditions (without applied external force, which changes the shape of the electrochemical cell). Moreover, as the mentioned membrane can work successfully, especially at lower currents, it is possible to apply it to other energy storage devices like aqueous batteries or pseudocapacitors, where lower currents than those of EDLCs are applicable. Furthermore, compared to typical glass fibre separators, this type of biopolymer membrane has excellent flexible properties and can be easily transferred to more industrial-type cells, like pouch cells or cylindrical cells.

## Figures and Tables

**Table 1:** Short literature review of the biopolymer membranes and their use in the aqueous EDLCs.

Ref.	Membrane	Electrolyte	Electrode Active Material	U [V]	ESR [Ω]	EDR [Ω]	C [F g^−1^]	E [Wh kg^−1^]	Longevity
[6]	Chitin/ Cellulose	1.0 mol L^−1^ lithium sulphate	AC Kynol ACC-507-20	0.8	1	1.7	90 *	2	20 k cycles, 5% capacitance drop
[28]	Cellulose	2.0 mol L^−1^ lithium acetate	AC Norti DLC Supra 30 AC Kynol ACC-507-20	0.8	5	15	20-25	2	10 k cycles, no observable drop
[33]	Cellulose nanofibrils	1.0 mol L^−1^ sodium sulphate	AC (not specified)	1.2	1–3	12–22	80–100 *	23–27 *	5 k cycles, up to 5% capacitance drop
[34]	Chitosan/Sodium alginate	2.0 mol L^−1^ lithium sulphate	AC Kynol ACC-507-20	1.6	1	3–5	125 *	8–10	1 k cycles, 10% capacitance drop
[35]	Chitosan/NaOH /glutaraldehyde	2.0 mol L^−1^ lithium acetate	AC Kynol ACC-507-20	0.8	1–3	5–10	100 *	2	10 k cycles, no observable drop
[36]	Chitin	2.0 mol L^−1^ lithium acetate	AC Kynol ACC-507-20	0.8	1	5	100 *	2	10 k cycles, no observable drop
[37]	Carboxylated chitosan	1.0 mol L^−1^ hydrochloric acid	AC Shenyang Kejing	0.9	1	8	40	3	(not specified)
[38]	Starch	1.0 mol L^−1^ sulphuric acid	AC (as prepared)	0.8	0.5	9–63	100–250 *	10–20 *	2 k cycles, 3% capacitance drop
[39]	Cellulose/Agarose	1.0 mol L^−1^ sulphuric acid 1.0 mol L^−1^ sodium sulphate	AC Kurary YP-80F	0.8	1–14	1–30	100–120 *	2	10 k cycles, 10% capacitance drop
[30]	Agar	0.5 mol L^−1^ potassium sulphate	AC Kynol ACC-507-20	1.6	1	2	80–110 *	7	10 k cycles, up to 8% capacitance drop
This work	Isinglass	1.0 mol L^−1^ sodium sulphate	AC Kynol ACC-507-20	1.6	2	7	25	8–10	10 k cycles, up to 5% capacitance drop
	Glass fibre	1.0 mol L^−1^ sodium sulphate	AC Kynol ACC-507-20	1.6	1	3	28	8–10	10 k cycles, up to 4% capacitance drop

AC—activated carbon; *—values presented per mass of one electrode.

## Data Availability

The datasets generated for this study are available on request to the corresponding author.
